# Integrated Host Genome Rumen Microbiome Metabolome Prediction of Low-Methane, High-Feed-Efficiency Sheep Under Climate Stress

**DOI:** 10.3390/ijms27219707

**Published:** 2026-10-29

**Authors:** Shahab Ur Rehman, Mohamed Tharwat, Aftab Shaukat, Abdulrahman A. Alkheraif, Rahmat Ali

**Affiliations:** 1 Laboratory of Metabolic Manipulation of Herbivorous Animal Nutrition, College of Animal Science and Technology, Yangzhou University, Yangzhou 225009, China; shahab@yzu.edu.cn; 2 Department of Animal Medicine, Faculty of Veterinary Medicine, Zagazig University, Zagazig 44519, Egypt; 3 Animal Science Division, Department of Modern Agriculture, College of Life and Environmental Sciences, Hunan University of Arts and Science, Changde 415000, China; dr.aftabshaukat@huas.edu.cn; 4 College of Veterinary Medicine, South China Agricultural University, Guangzhou 510642, China; 5 Department of Pathology and Laboratory Diagnosis, College of Veterinary Medicine, Qassim University, P.O. Box 6622, Buraidah 51452, Saudi Arabia

**Keywords:** enteric methane, residual feed intake, rumen microbiome, multi-omics prediction, genomic selection, heat stress, *Methanobrevibacter*, climate stress, sheep

## Abstract

Sheep are well adapted to marginal grazing systems and provide important products such as wool, milk and meat. However, microbial fermentation in the rumen and reticulum generates enteric methane, which contributes to greenhouse gas emissions from livestock production. Both methane emissions and feed efficiency show heritable variation, while host genetics can also shape rumen microbial composition through complex host–microbe interactions. This review explores how host genomics, rumen microbiome profiles, methanogenic archaeal communities, and rumen and blood metabolomics can be combined to identify biomarkers of low methane emissions and improved feed efficiency, particularly under environmental stress. Evidence suggests that methane production is repeatable and genetically influenced, and that rumen microbiome composition is associated with methane emission and feed efficiency, although current evidence does not show that specific microbial taxa directly cause these traits. Integrating host genotype with microbial and metabolic information may therefore improve statistical prediction of methane emissions and residual feed intake, which is not the same as identifying validated biomarkers or showing biological causation. Nevertheless, reliable microbial biomarkers have yet to be established, and validation across breeds, diets, and production environments remains limited. Heat stress adds further complexity by altering rumen fermentation, oxidative status, and microbial community structure, yet integrated omics research under climatic stress remains scarce. Future studies should prioritize longitudinal, multi-breed designs, standardized methane phenotyping, and coordinated multi-omics analyses to develop robust biomarkers for sustainable, low-emission sheep production.

## 1. Introduction

Sheep play an important role in utilizing grazing lands that are often unsuitable for crop production by converting fibrous plant materials into valuable products such as meat, milk and wool. However, this unique ability depends on rumen microbial fermentation, a process that also generates methane as a by-product. Enteric methane is an important component of the greenhouse gas footprint of sheep production [1]. Enteric methane is also a loss of dietary energy: part of the gross energy an animal eats leaves as methane instead of supporting maintenance or production. Lower methane output could therefore free more energy and nutrients for growth, wool, milk or meat, although this gain is not automatic and depends on where the saved energy goes [2]. Therefore, reducing enteric methane emissions in sheep while maintaining their productive efficiency has become an important breeding and management objective in several countries.

Two observations support selective breeding for lower methane emissions. First, methane traits in sheep are heritable and repeatable: Pinares-Patiño et al. [3] estimated heritabilities of 0.13 for methane yield (g CH_4_/kg dry-matter intake) and 0.29 for daily methane output in more than 1200 animals measured in respiration chambers. Second, animals differ in their rumen microbiome, and part of that variation is associated with host genetics [4,5]. Rumen microbial profiles can therefore be studied as phenotypes in their own right or as potential proxies for traits that are expensive to measure directly.

Feed efficiency adds another layer of complexity to methane-mitigation strategies. More efficient sheep generally eat less while maintaining the same level of production, which usually means lower absolute methane emissions [6]. Whether efficiency and low methane yield (methane per unit of feed) are actually the same target is harder to answer, and this review returns to that question several times. Climate stress complicates things further: rising heat load alters feed intake, rumen fermentation, oxidative stress physiology, and rumen microbiome composition and function [7,8]. Therefore, prediction models built under thermoneutral conditions may transfer poorly when applied to animals facing heat stress, restricted water, or low-quality forage.

No single data type is enough on its own to explain the biological variation behind methane emission and feed efficiency. Host genotype captures the heritable, transmissible part but explains only some of the phenotype. The microbiome is the working machinery of fermentation, though it is diet-sensitive and partly transient. Diet-driven shifts in rumen and gut microbiota that accompany changes in host metabolic and inflammatory phenotypes have also been reported in goats [9], although that work did not address methane. The metabolome shows the biochemical output, but only as a snapshot. This review proposes that these molecular layers contain complementary and partly independent information, and that their integration has shown potential to improve prediction of methane emission and feed efficiency in sheep, as demonstrated in proof-of-concept studies [10]. The remaining challenge is to develop robust multi-omics approaches that remain predictive under the diverse environmental conditions sheep experience, including climate-related stressors [11,12,13,14,15]. Earlier reviews have mostly treated host genomics, the rumen microbiome and metabolomics as separate lines of evidence. This review looks at the three together in sheep and asks how heat stress and other climate pressures change the links between them. Its objectives are to (i) summarize the evidence on heritability and repeatability of methane and feed-efficiency traits in sheep; (ii) assess what rumen microbiome, archaeome and metabolome profiles add to prediction of those traits; (iii) describe how far host genomic, microbial and metabolic data have actually been combined in sheep; and (iv) identify the gaps that heat stress introduces, so that a framework can be outlined for validated, climate-resilient biomarkers of low-methane, high-efficiency sheep.

## 2. Enteric Methane Emission in Sheep

**Figure 1:**
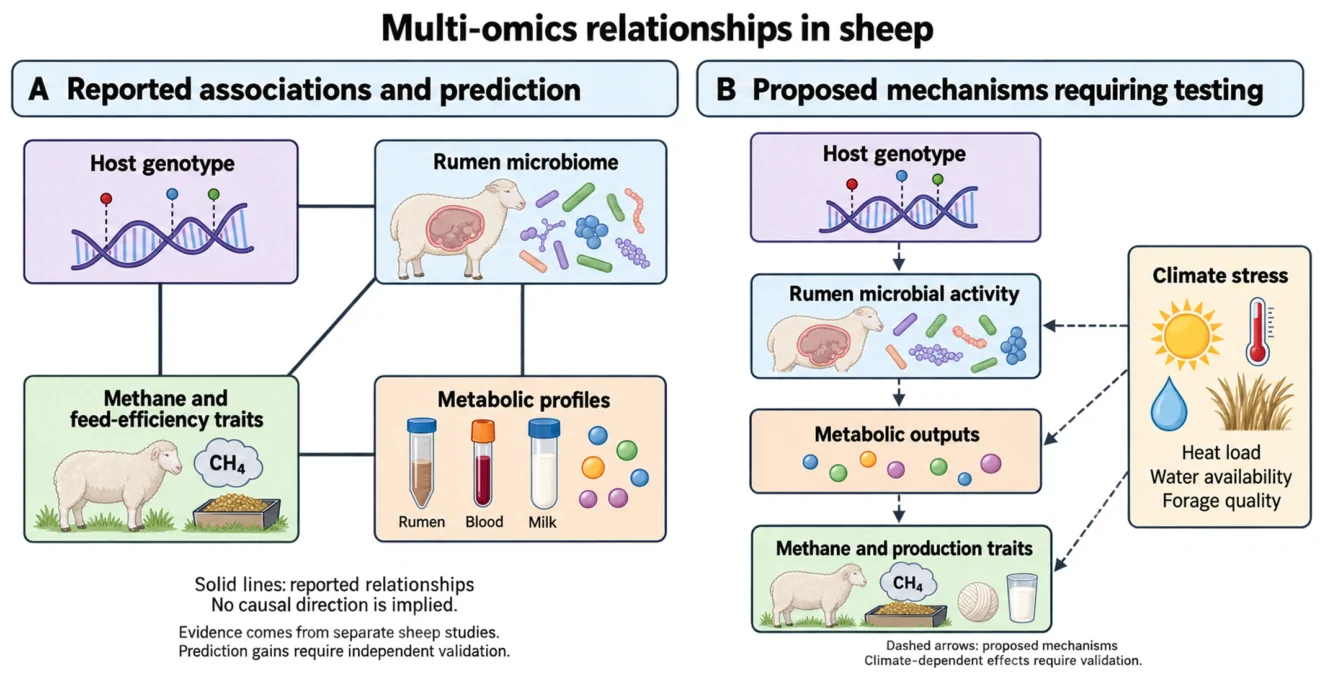
Evidence and hypotheses linking host genomics, rumen microbial profiles, metabolomics, and methane-related phenotypes in sheep. Panel (**A**) uses solid, undirected lines for reported statistical associations or predictive relationships; these lines do not establish causation. Panel (**B**) uses dashed arrows for proposed causal ordering and environmental modification that require testing. The evidence comes from different studies, rather than a single validated causal model (Section 4, Section 5, Section 6, Section 7 and Section 8). Climate-dependent prediction and genotype-by-environment effects remain research priorities (Section 7 and Section 10). In Panel (**A**), box colors identify the data layers: purple, host genotype; blue, rumen microbiome; orange, metabolic profiles; and green, methane and feed-efficiency traits. In Panel (**B**), the same colors apply, with cream indicating climate stressors (heat load, water availability, and forage quality). Supporting sheep studies include [3,4,10,20,21].

Methane is produced in the rumen by methanogenic archaea, which use hydrogen and carbon dioxide generated during microbial fermentation of feed. Methanogenesis acts as a hydrogen sink by converting CO_2_ and H_2_ into CH_4_, thereby maintaining rumen redox balance and preventing hydrogen accumulation that can disrupt microbial fermentation [16,17]. Methanogenesis is the main hydrogen sink in the rumen, but not the only one. Propionate formation, reductive acetogenesis, biohydrogenation of unsaturated fatty acids, and sulfate or nitrate reduction also take up hydrogen, so any intervention that suppresses methanogens should be checked for where the hydrogen goes instead. Methane output also depends on diet, hydrogen supply, microbial interactions and host physiology, not on methanogen numbers alone [17]. The dominant methanogenic archaeal groups are fairly consistent across animal hosts and geographic regions. The Global Rumen Census analyzed 742 rumen and camelid foregut samples from 32 host species across 35 countries and found that, despite wide variation in hosts and environments, a small number of archaeal groups consistently dominated, while protozoal communities varied more [18]. Malik et al. [19] combined metagenomic sequencing with the sulfur hexafluoride (SF_6_) tracer technique to characterize the archaeome of Indian sheep, finding that hydrogenotrophic methanogens predominated, with *Methanobrevibacter* as the dominant genus. The genetic potential for acetoclastic methanogenesis was limited, and methylotrophic methanogens made up only a minor part of the archaeal community [19]. Measured methane emission in that study averaged 19.7 g/day, a methane yield of 20.8 g/kg dry matter intake. This conservation across hosts matters for mitigation strategy: dominant archaeal groups are plausible targets, but archaeal taxonomic composition alone does not fully explain differences between high- and low-methane animals. Functional activity, hydrogen availability, microbial interactions, and host factors likely account for much of the remaining variation. Animal-related factors also shape the picture and are not covered in detail here: production type (meat, wool or dairy), age and physiological stage (growing lambs versus mature ewes or rams), and pregnancy or lactation. These factors should be considered when interpreting methane output and intensity. Section 7, “Climate Stress in Sheep,” discusses heat-related changes in intake, rumen fermentation, and host physiology; interactions with animal class require further study. Table 1 lists the main sheep studies on methane, the rumen microbiome and feed efficiency that underpin this review, and Figure 1 shows the framework used to organize it.

**Table 1:** Selected recent sheep and ruminant studies on methane emission, rumen microbiome, and feed efficiency.

System/Animal Model	Study Design and Methods	Key Finding Relevant to This Review	References
Sheep, >1225 animals	Respiration chambers and quantitative genetic analysis	Methane traits were heritable and repeatable, with heritability estimates of 0.13 for methane yield and 0.29 for daily methane emissions.	[3]
Low- and high-methane sheep	Metagenomic, metatranscriptomic and 16S rRNA profiling	Low-methane sheep had rumen communities enriched with lactate-forming *Sharpea*, with approximately 24% lower methane production based on stoichiometric estimates.	[20]
Sheep	Metagenomic/metatranscriptomic analysis and co-culture experiments	High methane yield was associated with bacterial type III secretion genes and the presence of *Succinivibrio*.	[22]
Merino sheep	Automated feeders, portable accumulation chambers and genetic analysis	Intake and residual feed intake showed strong genetic correlations with methane traits, ranging from 0.76 to 0.90; CO_2_ was also identified as a potential proxy for intake.	[6]
Sheep, including lambs and ewes	Portable accumulation chambers compared with respiration chambers	Methane measured using portable accumulation chambers showed heritability of 0.19–0.20; lamb–ewe genetic correlation was 0.99, while PAC–chamber correlations ranged from 0.62 to 0.67.	[23]
Sheep, 99 animals	Integrated genome, metabolome and microbiome BLUP prediction	In a 99-sheep proof of concept, combined models gave modest improvements over genomic prediction alone; independent validation across populations and environments was not established.	[10]
Indian sheep	Metagenomic analysis and SF_6_ tracer technique	Hydrogenotrophic *Methanobrevibacter* dominated the methanogen community; methane output was 19.7 g/day and 20.8 g/kg DMI.	[19]
Sheep; meta-analysis of 13 studies	Random-effects meta-analysis	Heritability estimates for gas traits ranged from 0.137 to 0.250, while the genetic correlation between methane and residual feed intake was 0.408.	[24]
Sheep, 1200 ewes	Host genotype data combined with RE-RRS metagenomic profiling	Combined host and microbial models explained substantial phenotypic variation; variance explained is distinct from accuracy in an independent population.	[4]
Sheep, 1708 animals	RE-RRS metagenomic profiling across environments	Some rumen genus abundances were heritable and repeatable; however, diet, age and time off feed had stronger effects on microbial composition.	[5]
Sheep	Latin square design, portable accumulation chambers and 16S rRNA profiling	Sward type influenced methane emissions, nitrogen output and the abundance of *Methanobrevibacter* and *Methanosphaera*.	[25]
Sheep, 400 ewes from divergent lines	Rumen traits and milk-based proxy measurements	The low-methane line showed a lower acetate-to-propionate ratio; milk-based proxies were heritable and genetically correlated with methane emissions.	[21]
Sheep, 4585 lambs	RE-RRS metagenomic profiling and SNP analysis	Microbiome-predicted methane was genetically correlated with PAC-measured methane, with correlations ranging from 0.64 to 0.75.	[26]

Abbreviations: PAC, portable accumulation chamber; RE-RRS, restriction-enzyme reduced-representation sequencing; RFI, residual feed intake; DMI, dry-matter intake; BLUP, best linear unbiased prediction; SNP, single-nucleotide polymorphism.

**Figure 2:**
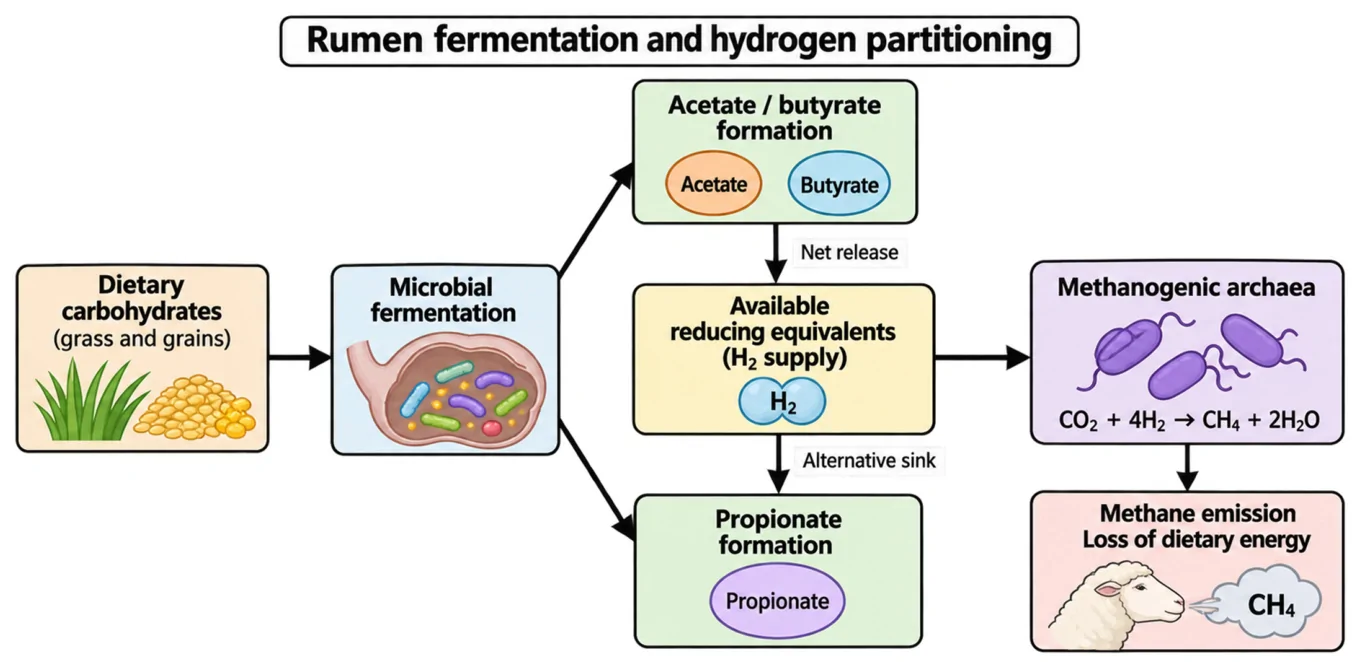
Mechanistic links among diet-driven rumen fermentation, microbial hydrogen partitioning, methane production, and host energy efficiency. Dietary carbohydrates are fermented to acetate, propionate, butyrate, and lactate. Acetate and butyrate formation releases reducing equivalents, whereas propionate formation provides an alternative route for their disposal. Hydrogen availability and competing microbial pathways influence methane formation and dietary energy loss. Redirecting fermentation may reduce methane, but benefits to animal performance require direct evaluation.

Variation in bacterial fermentation and hydrogen supply can contribute to methane differences even when the dominant archaeal groups are similar [17,27]. The volatile fatty acid (VFA) profile matters here: fermentation to acetate and butyrate releases hydrogen, while fermentation to propionate consumes it, so a rumen that channels carbon toward propionate leaves less hydrogen available for methanogenesis. One clear example linking rumen microbial function to methane variation in sheep comes from Kamke et al. [20], who found the lactate-producing genus *Sharpea* enriched in low-methane-yield sheep. They proposed that increased lactate production, and its subsequent conversion to butyrate, could redirect reducing equivalents away from methanogenesis, lowering methane production per unit of hexose fermented compared with acetate-dominated fermentation. Stoichiometric analysis suggested this pathway could cut methane production by roughly 24%. A similar principle underlies dietary strategies that redirect fermentation toward propionate, another major hydrogen sink. Sato et al. [28], using in vitro cultures inoculated with sheep rumen fluid, found that calcium salts of linseed oil fatty acids increased propionate production and cut methane emissions by 38 to 63%, accompanied by shifts in propionate-associated bacterial genera, including *Succinivibrio*, *Succiniclasticum*, *Selenomonas*, and *Megasphaera*. The acetate-propionate balance also connects methanogenesis to how much dietary energy the host retains. Methane represents feed energy lost as gas rather than captured by the animal, so shifting fermentation toward propionate may both improve energy retention and reduce emissions. This gives a biological basis for expecting low methane emissions and high feed efficiency to go together. But the relationship is not guaranteed: acetate is also an important energy substrate and a precursor for milk fat and body fat, so indiscriminately suppressing acetate production could hurt animal performance. The goal is not simply to minimize methane, but to reduce emissions while maintaining or improving productive output. For that reason, methane yield (methane per unit of feed intake) and methane intensity (methane per unit of product) are usually more informative than absolute emissions alone (Figure 2). Lower methane does not always translate into better growth, wool or milk output, so mitigation should be judged against production and not against emissions alone.

## 3. Feed Efficiency and Low-Methane Phenotypes

Residual feed intake (RFI) is the most commonly used measure of feed efficiency in ruminant research. It is calculated as the difference between an animal’s actual feed intake and the amount predicted from its body size and production level. Animals with low RFI values consume less feed than expected while maintaining comparable production and are therefore considered more efficient. Because feed intake influences hydrogen generation and methane formation in the rumen, animals with low RFI may also produce less methane. In Merino sheep, Paganoni et al. [6] reported strong genetic correlations between feed intake, RFI, and methane emission, suggesting that selection for improved feed efficiency could contribute to reduced methane production. However, a meta-analysis by Ghavi Hossein-Zadeh [24], including 13 studies published from 2010 to 2022, estimated more moderate genetic correlations between methane production and RFI (0.408) and between methane production and feed intake (0.598), with methane emission heritability estimates ranging from 0.137 to 0.250. Because RFI is the residual from a regression on body size and production, it compares animals of different size and output more fairly than raw intake does. Efficiency rankings measured under thermoneutral conditions may not hold under heat stress, which lowers feed intake and changes rumen fermentation [7,8] (Section 7, “Climate Stress in Sheep”).

However, these correlations mainly describe methane production, which is strongly influenced by feed intake. When methane emission is adjusted for intake, the remaining variation is smaller and shows weaker relationships with production traits. Paganoni et al. [6] reported that methane emission corrected for feed intake had weak and generally nonsignificant genetic correlations with most production traits, except for a modest favorable association with fleece weight. Thus, selecting animals that consume less feed for a given level of production can reduce total methane output. In contrast, selection for lower methane yield represents a distinct breeding objective and may not directly improve growth or wool production. Recognizing this distinction is essential when developing breeding strategies for low-emission sheep. In practice, identifying low-methane animals still depends largely on direct methane measurements. However, the cost and labor involved have encouraged the development of indirect indicators and early-life predictors. One study showed that gas-related traits could be measured after weaning and identified carbon dioxide—which is easier and less expensive to measure than methane—as a useful indicator of feed intake [6]. Another study extended this proxy-based approach to lactating ewes divergently selected for methane emissions. Ewes from the low-methane line produced milk containing more polyunsaturated and branched-chain fatty acids and less saturated fat. Proxy methane traits derived from milk or rumen composition were heritable, with estimates ranging from 0.12 to 0.36, and showed genetic correlations of 0.52–0.71 with measured methane. Although somewhat unexpected, milk-based proxies performed better than rumen VFA profiles, highlighting their potential as practical indicators for indirect selection [21]. Methane production, methane yield and feed efficiency are related but separate traits, and they should not be treated as interchangeable selection objectives.

Productivity traits beyond growth and wool may also be linked to the microbiome, although the available evidence remains limited and is derived largely from intervention studies. Dou et al. [29], for example, found that supplementation with *Clostridium butyricum* altered rumen and intestinal microbial communities and was associated with improved skeletal muscle development and meat quality in lambs. These effects may involve a gut–muscle axis associated with increased butyrate and valerate production [29]. Although such findings provide mechanistic insight rather than directly applicable selection markers, they demonstrate that fermentation-related traits associated with methane production and feed efficiency are closely connected to downstream outcomes such as meat and milk production [11,30]. The intervention affected both the microbiome and production outcomes; this does not establish that microbial changes mediated the production response.

## 4. Host Genome Contribution to Methane and Feed Efficiency

Evidence for host genetic contributions to methane emissions in sheep comes primarily from low-to-moderate heritability estimates. Pinares-Patiño et al. [3] estimated heritabilities of 0.29 for daily methane production and 0.13 for methane yield, with corresponding repeatabilities of 0.55 and 0.26 for measurements taken two weeks apart. Using portable accumulation chambers in grazing sheep, Jonker et al. [23] reported methane heritabilities of 0.19 in lambs and 0.20 in ewes, together with a genetic correlation of 0.99 between lamb and ewe measurements. These findings support the use of early-life methane phenotypes in genetic evaluation. A meta-analysis by Ghavi Hossein-Zadeh [24] reported average heritabilities of 0.137–0.250 for methane-related traits. Although progress from selection may be gradual, these estimates support genetic improvement when scalable phenotyping is available. Daily methane production (g/day) and methane yield (g/kg dry-matter intake) are distinct traits; their heritabilities should be interpreted separately.

The host can influence rumen microbial community structure through genetic and physiological interactions, providing a conceptual link between host genomics and the microbiome. Similar nutritional and host effects on rumen and gut microbiota are reported in goats, where diet and supplement studies link microbial shifts to host metabolic and inflammatory traits [31]. These studies are neither about methane nor about sheep, but they illustrate the general principle. Several mechanisms have been proposed but remain largely untested: host control of rumen epithelial structure and absorption, saliva composition and buffering, mucosal immunity and antimicrobial peptides, rumen pH and motility, and heritable differences in feeding behavior and intake rate. Telling these apart would need studies that pair host genotype with rumen epithelial transcriptomics, not microbiome composition alone. Hess et al. [5] analyzed 4479 rumen metagenomic samples from 1708 sheep and found that the abundance of several microbial genera was heritable and repeatable, with some effects remaining detectable across dietary conditions [5]. Nevertheless, overall microbial community composition was still mostly shaped by environmental factors, including diet, age, and time off feed [5]. Cattle studies also report heritable microbial features associated with host traits, providing cross-species context rather than direct sheep evidence [15,32,33]. The heritable fraction makes microbiome-informed selection a plausible strategy. If host genetics consistently shifts the microbial community toward a low-methane configuration, selection targeting either the host genome or heritable microbial features could influence methane phenotypes in subsequent generations.

Genomic prediction converts heritable variation into estimated breeding values usable for selection. Ross et al. [10] carried out genomic prediction of a residual methane phenotype in 99 animals and reported prediction accuracies ranging from 0.058 to 0.220 using genomic relationships alone, depending on the measurement time point [10]. These modest accuracies were expected given the small reference population and moderate heritability of methane-related traits, and they set a baseline against which the value of additional biological information can be judged. Hess et al. [4] built on this approach by integrating host genotype with rumen metagenomic profiles from 1200 ewes, reporting substantially better prediction of methane emission and residual feed intake than genomic information alone achieved, especially when microbial profiles from early life were included [4].

A major limitation in sheep is the limited resolution of causal genetic variation underlying methane emission and feed efficiency. Current heritability estimates and genomic prediction methods rely mostly on genome-wide relationships rather than identified quantitative trait loci or functionally characterized genes involved in metabolic and immune regulation. Candidate gene studies and quantitative trait locus (QTL) mapping for methane-related traits in sheep are less developed than in dairy cattle, and much of what is known about the genomic architecture of these traits comes from cattle research. Associations between specific host genes and methane phenotypes in sheep should therefore be interpreted cautiously until more sheep-specific genomic studies exist. Resolving these causal relationships is an important research gap, one that needs to be closed to develop more mechanistically informed host-genome-based selection strategies. To date, the causal genes behind methane emission and feed efficiency in sheep remain largely unidentified, and the evidence is mainly genomic association.

## 5. Rumen Microbiome, Archaeome, and Methane Phenotypes

The rumen microbiome is better understood through its functional capacity than through taxonomic composition alone, since different microbial communities can produce similar methane phenotypes if they converge on comparable fermentation strategies. Hydrogen availability and use are central to the mechanism linking microbial activity with methane production: communities that generate less hydrogen, or that redirect reducing equivalents toward alternative pathways, leave less substrate available for methanogenesis. The archaeal community in sheep varies little across animals. Malik et al. [19] found that hydrogenotrophic *Methanobrevibacter* species dominate the sheep archaeome, with methane formation supported mainly by the CO_2_-reduction pathway and a smaller contribution from methylotrophic methanogenesis [19]. Similarly, Xie et al. [34] reported that the *Methanobrevibacter gottschalkii* clade accounted for approximately 60% of methanogens in Hu sheep [34]. Because archaeal taxonomic differences alone may not fully explain variation in methane emission, the more informative predictive signals may arise from bacterial communities and functional pathways that regulate hydrogen production, transfer, and utilization.

Contrasting bacterial patterns illustrate why associations need to be interpreted at the level of species, strains, and experimental context. Kamke et al. [20] identified a *Sharpea*-enriched microbiome in low-methane-yield sheep, with metagenomic and metatranscriptomic evidence consistent with lactate formation and utilization. Their estimated reduction in methane was based on fermentation stoichiometry, not an intervention establishing that *Sharpea* alone lowers emissions. In a separate study, Kamke et al. [22] associated bacterial type III secretion genes with higher methane yield. In co-culture, *Succinivibrio dextrinosolvens* strain H5 stimulated one methanogen and inhibited another, demonstrating strain-specific interactions under laboratory conditions. Sato et al. [28] found changes in propionate-associated genera alongside reduced methane in batch cultures inoculated with sheep rumen fluid. These findings do not establish that the genera predict methane consistently in live sheep. Table 2 distinguishes direct sheep associations, sheep-derived in vitro findings, and broader ruminant evidence. Culture studies confirm lactate production by *Sharpea* and *Kandleria* [35], but the contribution of individual taxa to methane phenotypes in vivo still requires functional validation.

*Prevotella*, the most abundant bacterial genus in many sheep rumen datasets, is harder to place. It is metabolically versatile, contributes to propionate production through succinate, and shifts in abundance under dietary and heat-stress interventions [8], but its sheer diversity means genus-level abundance is a blunt predictor of methane. This is a recurring problem. Taxonomic resolution at the genus level often hides functionally important strain-level differences, which is part of why reference-free metagenome profiling has gained ground over reference-based or 16S approaches for prediction [4,36]. Genus-level labels also group species and strains that differ in function, which limits their value as markers.

Protozoa and fungi are the least resolved components in the sheep methane literature. Protozoa transfer hydrogen to methanogens and associate with them physically, so defaunation lowers methane, and several plant secondary metabolites reduce methane partly by suppressing protozoa [37]. Mohapatra et al. [38] observed a 28.4% reduction in methane production in growing sheep fed seaweed biowaste, together with decreased protozoal abundance and increased sulfate-reducing bacteria, suggesting altered hydrogen competition between methanogens and alternative microbial pathways [38]. Anaerobic fungi degrade fiber and generate hydrogen, so they belong to the hydrogen economy, yet their role in between-animal methane variation in sheep remains poorly characterized. Any biomarker panel built only on bacteria and archaea is therefore incomplete, which is a genuine limitation of current predictive work. Both groups also take part in hydrogen transfer to methanogens, so they should be included in future profiling. Heat stress also changes rumen microbial composition [7,8], so the taxa in Table 2 may behave differently under thermal load (Section 7).

**Table 2:** Candidate microbial indicators and the experimental context of evidence relevant to sheep methane phenotypes.

Taxon	Domain/Microbial Type	Observed Pattern	Functional Interpretation and Limits	Evidence Setting and References
*Sharpea* spp.	Bacteria	Low methane	Lactate production is supported by culture studies; community-level lactate utilization may alter hydrogen partitioning. Taxon-specific methane reduction in vivo is unproven.	Direct sheep association and meta-omics [20]; isolate culture evidence [35].
*Succiniclasticum*	Bacteria	Propionate-associated; lower methane in batch culture	Succinate-to-propionate conversion is a proposed explanation; taxon-specific methane effects were not isolated.	Sheep-rumen in vitro study [28]; heat-stressed lamb supplementation study without methane validation [8].
*Selenomonas*	Bacteria	Propionate-associated; lower methane in batch culture	Propionate-related metabolism may redirect reducing equivalents; abundance changes do not establish causation.	Sheep-rumen in vitro study [28]; not a validated live-sheep methane marker.
*Succinivibrio*	Bacteria	Context-dependent	Co-culture effects differ by methanogen strain; a uniform high- or low-methane function cannot be assigned at genus level.	Sheep gene/transcript associations and isolate co-culture [22]; sheep-rumen in vitro study [28].
*Prevotella* spp.	Bacteria	Context-dependent	Versatile fermenter involved in succinate and propionate pathways; genus-level classification is too broad for a consistent methane signal.	Heat-stressed lamb supplementation study [8]; methane-marker performance not tested.
*Methanobrevibacter*	Archaea	Dominant methanogen; abundance is not a validated high-methane marker	Dominant hydrogenotrophic methanogen using hydrogen and carbon dioxide to produce methane.	Direct sheep archaeome and intervention studies [19,34]; taxonomy alone does not establish predictive value.
*Methanosphaera*	Archaea	Variable	Methylotrophic methanogenesis; abundance may shift with diet, heat stress or environmental conditions.	Direct sheep dietary and heat-stress intervention studies [8,25]; no independently validated methane marker.
Rumen protozoa	Protozoa	Often associated with methanogenesis	Promote interspecies hydrogen transfer to methanogens to support methane production.	Broader ruminant synthesis [37] is cross-species context; sheep dietary intervention [38] does not isolate protozoal causation.
Sulfate-reducing bacteria	Bacteria	Increased abundance alongside lower methane in a dietary trial	Compete with methanogens for hydrogen, potentially reducing methane formation.	Direct sheep intervention [38]; hydrogen competition is a proposed mechanism, not a taxon-specific causal test.

## 6. Rumen and Blood Metabolomics

Metabolomic profiles describe biochemical processes that may help explain variation in methane-related phenotypes. Bilton et al. [21] reported a lower ruminal acetate-to-propionate ratio in ewes from a low-methane selection line. Ross et al. [10] used metabolite-based relationship matrices to predict residual methane in 99 sheep, reporting accuracies of 0.254 and 0.132 at two measurement time points. These results support further evaluation of the rumen metabolome as a predictive layer, but do not establish performance in independent populations. Table 3 distinguishes candidate metabolites from protein stress markers; none should be treated as an independently validated methane biomarker on the basis of these studies alone.

Peripheral lipid metabolites provide an additional advantage because they can be measured without direct rumen sampling. Milk from low-methane ewes contained higher proportions of polyunsaturated and branched-chain fatty acids and lower levels of total saturated fatty acids. Because branched-chain fatty acids originate partly from microbial membrane components and branched-chain amino acid metabolism, they may serve as indirect indicators of rumen microbial activity and fermentation patterns. In that study, milk-based proxies showed greater promise than rumen VFA measurements [21]; this does not establish their general superiority across populations. Beyond VFAs and fatty acids, metabolomic evidence in sheep remains limited. Amino acids, bile acids, and energy- or oxidative-stress-related metabolites have been proposed as potential biomarkers of feed efficiency and physiological status in ruminants, but sheep-specific studies linking defined blood metabolite profiles to methane emission are scarce, and most current understanding comes from cattle research [39,40,41]. The strongest evidence for metabolomic responses to environmental stress in sheep comes not from methane research but from climate studies. Untargeted metabolomics in heat-stressed Hu rams showed substantial reorganization of the rumen and seminal metabolome, including changes in cortisol- and steroid-related pathways and reductions in metabolites such as kaempferol and L-tryptophan, both associated with reproductive function [7]. This confirms that the rumen metabolome responds to heat stress, but whether these changes connect to methane production in the same animals is still unknown. Amino acids and oxidative-stress-related compounds may add useful biological information, but their value as methane predictors in sheep still needs validation. At present, integrating blood metabolomic profiles with methane and feed-efficiency phenotypes in sheep remains a research objective rather than an established practice. Metabolomic markers are promising predictors but have not been validated for practical methane prediction in sheep, and sheep-specific studies are few, so conclusions borrowed from cattle should be treated cautiously. Metabolite shifts seen under heat stress (Section 7) show that these candidate markers depend on environmental context.

## 7. Climate Stress in Sheep

Climate stress affects the rumen through several interconnected pathways, including reductions and changes in feed intake, direct physiological responses to elevated temperatures, declining forage quality and availability, and limited access to water. Among these stressors, heat stress has been studied most extensively in sheep using omics-based approaches. These studies have identified molecular and microbial changes associated with altered physiological responses and daily metabolic patterns. Heat stress activates host oxidative-stress pathways and heat-shock responses, with adverse consequences for animal health, welfare, and productivity. Su et al. [8] studied 240 fattening lambs at a temperature–humidity index of 79.7 and evaluated the effects of herbal supplementation on physiological and rumen microbial responses. Heat-stressed control lambs had increased serum concentrations of heat shock proteins (HSP60, HSP70, and HSP90), together with altered antioxidant enzyme activity. The most effective supplement reduced these stress markers, improved growth performance, and modified the rumen microbial community by increasing the abundance of *Prevotella* and altering populations of *Succiniclasticum* and the methanogen *Methanosphaera* [8].

Similarly, supplementation with curcumin nanomicelles in heat-stressed lambs improved several measures of rumen fermentation, including total VFA production, acetate and propionate levels, and microbial protein synthesis. These changes were associated with improved growth performance and nutrient digestibility under heat-stress conditions [42]. Collectively, these studies demonstrate that heat stress disrupts rumen function and that nutritional interventions may improve fermentation under these conditions. However, the available evidence comes mainly from short-term feeding trials and does not establish how heat stress affects genetic variation or genotype-by-environment interactions for methane-emission traits.

Thus, although the connection between climate stress and methane intensity is mechanistically plausible, it remains poorly characterized in sheep. Heat-related reductions in intake and productivity could affect methane output and intensity differently; the supplementation studies above did not directly test this relationship. Drought, feed scarcity, poor forage quality and water restriction, arguably the more important climate pressures for extensive sheep systems, are barely represented in the rumen-omics literature at all. This is a substantial gap, because a low-methane genotype identified in a well-fed temperate flock may not keep its advantage, or its microbiome configuration, in a hot, feed-restricted environment. The stability of multi-omics predictions across climate conditions is essentially untested in sheep [4,7,10]. Integrated rumen metabolomics and metataxonomics in heat-stressed Korean native goats provide comparative evidence, but do not validate sheep methane predictions [43].

## 8. Multi-Omics Integration

The sheep proof-of-concept study by Ross et al. [10] combined genomic, rumen metabolomic, and microbiome information from 99 animals measured over three years. Genomic prediction accuracies ranged from 0.058 to 0.220. Adding metabolomic information improved accuracy from 0.201 to 0.274 at one time point and from 0.081 to 0.158 at another; adding microbiome information improved one comparison from 0.216 to 0.247. These modest gains suggest complementary predictive information, but the small population and study-specific validation do not establish performance across independent breeds, diets, or climates. Table 4 summarizes the data layers, methods, and limitations. Related goat studies provide cross-species context: Zhou et al. [44] combined microbial, proteomic, and metabolomic analyses during *Enterococcus faecalis* JM263 supplementation in high-concentrate-fed goats, while Zhou and Shen [45] examined cecropin supplementation, rumen fermentation, microbiota, and host immune and antioxidant responses. These are goat intervention studies of nutrition and health, not evidence that either intervention reduces methane in sheep.

Hess et al. [4] analyzed 3139 rumen samples from 1200 ewes using restriction-enzyme reduced-representation sequencing (RE-RRS) and combined host genomic and microbial information to predict methane emission, residual feed intake, and other traits. Reference-free microbial profiles generally performed better than reference-based profiles in that study. A separate analysis by Hess et al. [5] characterized environmental and genetic influences on 4479 samples from 1708 sheep. Bilton et al. [26] extended microbiome-based methane prediction to 4585 lambs, reporting genetic correlations of 0.75 with daily methane production and 0.64 with methane ratio. These correlations support the use of microbial profiles as candidate proxy traits, but are not interchangeable with individual prediction accuracy. Bilton et al. [21] separately evaluated milk fatty acids and mid-infrared spectroscopy as methane proxies in lactating ewes. Transferability across breeds and production environments remains a key validation requirement.

Most established sheep multi-omics prediction studies use mixed models and relationship matrices [4,10]. More recently, Alemu et al. [46] combined microbial principal components with host genomic information in a neural-network genomic best linear unbiased prediction (GBLUP) model. For methane emissions, accuracy increased from 0.09 to 0.30 in a train–test evaluation and from 0.15 to 0.27 in five-fold cross-validation. The improvement was trait-dependent and did not establish performance under climate stress. genome-wide association studies (GWAS), metagenomics, metatranscriptomics, and metabolomics remain complementary approaches for studying methane-related biology [16]; their contribution to prediction should be evaluated against transparent baselines.

A separate challenge is distinguishing predictive dependence from biological mediation. Genomic, microbial, and metabolic features are correlated, and models should account for that dependence. Host genetic effects on microbial composition and microbial contributions to metabolite profiles provide plausible hypotheses, but a fitted predictive model does not establish the direction of causation. Structural equation or mediation models may be useful for testing explicitly stated hypotheses when their assumptions and study design are appropriate. Longitudinal sampling and targeted interventions are needed to strengthen causal interpretation.

## 9. Predictive Modelling for Climate-Smart Sheep

A predictive framework for climate-smart sheep production should integrate biological and environmental information to identify animals with low methane emissions, high feed efficiency, and improved resilience to climate stress using measurements that can be collected at population scale. Based on current evidence, the major input categories include host genomic information (single-nucleotide polymorphism [SNP] genotypes); rumen microbial profiles, particularly scalable approaches such as RE-RRS; targeted metabolomic measurements, including rumen VFA profiles and, where available, milk fatty-acid profiles; production and methane-related phenotypes; and environmental variables describing thermal load, diet, and feed availability [4,5].

An effective modeling framework would ideally combine interpretable statistical approaches with flexible prediction methods. A mixed-model foundation, such as GBLUP incorporating genomic, microbial, and metabolomic relationship matrices, yields estimates of variance components and breeding values. Machine-learning methods can model nonlinear interactions, but their benefit depends on the trait, sample size, and validation design [46]. These flexible methods can overfit. Sheep multi-omics datasets usually hold hundreds to a few thousand animals but tens of thousands of microbial or metabolite features. Without cross-validation, held-out test sets and regularization, gains from random forests, gradient boosting or deep learning may reflect the training population rather than real generalizable predictive information. Such models should be compared with a simple linear mixed-model baseline before the extra complexity is accepted. Environmental variables should be built into these models explicitly, to estimate genotype-by-environment interactions rather than treat environmental variation as unexplained noise. That distinction, whether environmental context is modeled or ignored, separates conventional selection models from climate-resilient prediction frameworks.

**Figure 3:**
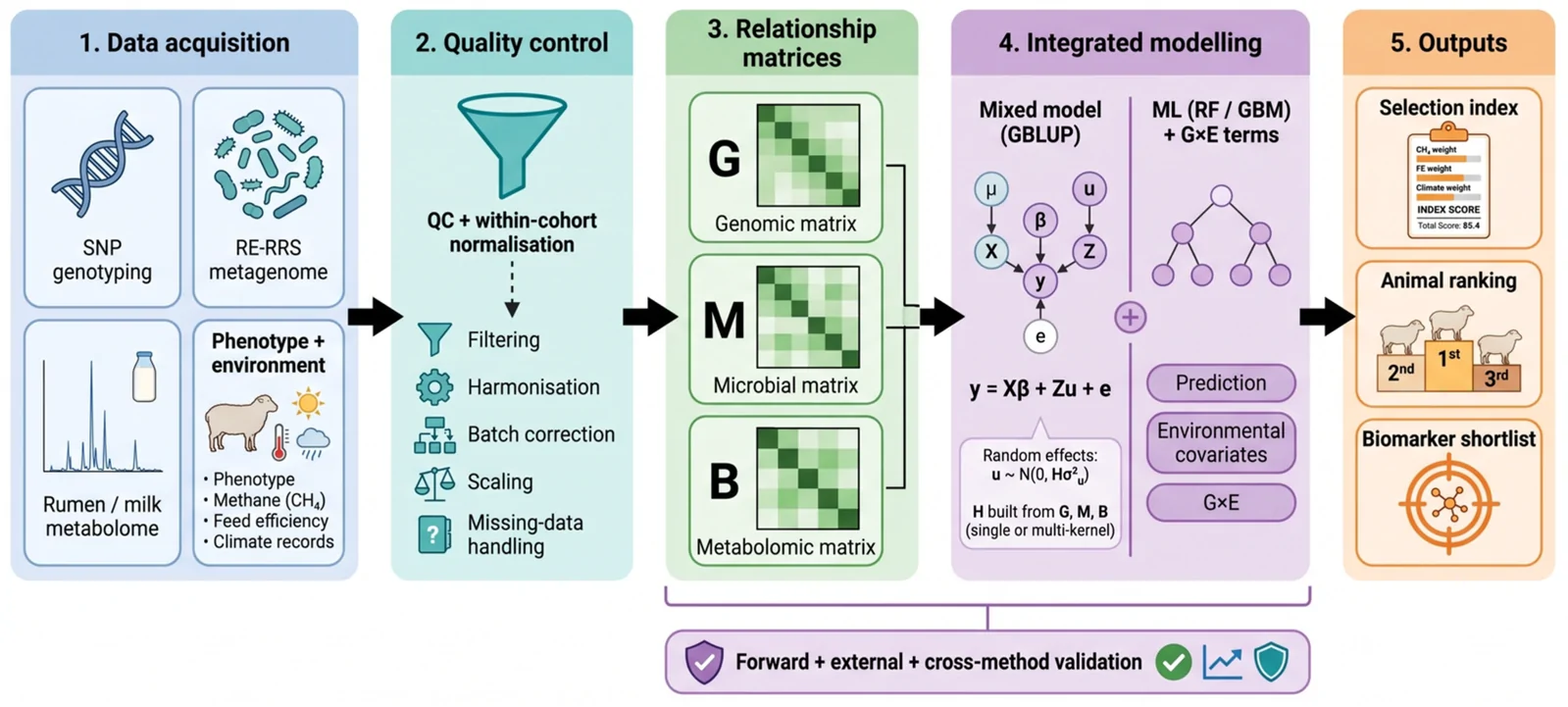
The pipeline integrates five data sources: SNP genotypes, rumen microbiome profiles, rumen and milk metabolomics, phenotypic records, and environmental data. Before modeling, the raw data are harmonized across sources, corrected for batch effects, scaled, and screened for missing values. The processed data are then used to construct genomic, microbial, and metabolomic relationship matrices. Each matrix is incorporated into mixed models and machine-learning algorithms together with environmental covariates and genotype-by-environment interaction terms. The resulting predictions are used to generate selection indices, rank animals, and identify candidate biomarkers. Forward, external, and cross-method validation are incorporated to assess whether model performance extends beyond the training data and remains applicable under field conditions. The five colored panels represent the pipeline stages, and thick black arrows show the direction of data flow from one stage to the next. Lines from the three relationship matrices converge on the integrated-modeling stage, and the lower bracket indicates that validation is applied across the matrix-construction and modeling stages. The index weights and score shown in the outputs panel are illustrative only. Abbreviations: SNP, single-nucleotide polymorphism; RE-RRS, restriction-enzyme reduced-representation sequencing; QC, quality control; G, genomic relationship matrix; M, microbial relationship matrix; B, metabolomic relationship matrix; GBLUP, genomic best linear unbiased prediction; ML, machine learning; RF, random forest; GBM, gradient boosting machine; G × E, genotype-by-environment interaction; FE, feed efficiency.

Validation remains a major obstacle to practical implementation. Hess et al. [5] showed that rumen profiles vary with biological and environmental factors, while Jonker et al. [23] reported only moderate genetic correlations between methane measurements obtained with portable accumulation chambers and respiration chambers. Models therefore need testing across measurement platforms, diets, breeds, and climatic environments. Forward evaluation in later cohorts and external validation in separate breeds or regions should be distinguished from random cross-validation within the same population. The proposed framework links methane and production objectives with candidate biomarkers for further testing (Figure 3; Table 5). Practical use requires evidence that predictions remain accurate in the intended population and production setting.

## 10. Methodological Challenges

Interpretation of methane-related traits depends strongly on the measurement method used, as different approaches capture the phenotype with varying levels of accuracy, throughput, and environmental relevance. Respiration chambers remain the reference standard and have generated many of the foundational methane heritability estimates in sheep; however, their high cost, limited throughput, and potential effects on animal behavior and feed intake restrict their application in large-scale studies [3]. The sulfur hexafluoride (SF_6_) tracer technique allows methane emissions to be measured under more natural grazing conditions and has been used in studies of rumen archaeal communities in sheep [19]. Portable accumulation chambers (PAC) offer a practical balance among accuracy, cost, and throughput and are therefore widely used for genetic evaluation. Nevertheless, methane measurements obtained using PAC and respiration chambers have only moderate genetic correlations of approximately 0.62–0.67. Consequently, breeding values derived from one method may not transfer fully to another measurement system [23]. Spot-sampling systems such as GreenFeed have been used more extensively in cattle, particularly for large-scale methane monitoring [47,48]. Given the limitations of direct measurement, indirect indicators such as milk fatty-acid profiles, mid-infrared spectroscopy, and rumen microbial profiles are receiving increasing attention. Their predictive reliability, however, depends on the quality and consistency of the reference methane measurements used for calibration [21,26].

Sampling design and analytical decisions also shape downstream biological interpretations. Diet is one of the strongest determinants of rumen microbial community composition; therefore, comparisons across studies conducted under different dietary conditions may be confounded unless diet is carefully controlled or incorporated into the statistical model [4]. Because diet shifts microbial composition so strongly, microbial biomarkers found under one diet may not transfer to another. Time off feed and sampling time must also be considered. Because heat stress can alter the daily dynamics of the rumen microbiome, sampling at a single fixed time may bias comparisons between heat-stressed and non-stressed animals [7]. Breed effects are also likely to be important but remain poorly characterized because most sheep multi-omics datasets come from a limited number of research populations, particularly in New Zealand and Australia. At the molecular level, sequencing depth and analytical strategy—including reference-based and reference-free profiling affect which microbial signals can be detected [4,26]. Without appropriate normalization and statistical correction, batch effects and cohort structure may be misinterpreted as biological variation [26]. Metabolomic findings are similarly influenced by the analytical platform because nuclear magnetic resonance (NMR) and mass spectrometry (MS) provide different coverage of the metabolite landscape.

## 11. Future Research Framework

Addressing current knowledge gaps will require a framework that moves beyond single-flock, cross-sectional studies toward longitudinal and multi-breed designs capable of capturing variation across environments and production systems.

Design and animals. Future studies should include prospective cohorts representing contrasting breeds and production systems, such as temperate wool breeds, meat breeds, and heat-adapted breeds, monitored across seasons that include defined periods of heat or nutritional stress. Given the moderate heritability of methane-related traits (0.13–0.29), effective genomic and microbiome prediction will require large reference populations, potentially involving thousands of animals, as illustrated by recent large-scale sheep studies. Selection outcomes should also be checked against maternal and health traits [3,4,5,21,24,26,49].

Climate-stress exposure. Climate challenges should be incorporated as defined experimental factors rather than relying only on natural variation. Continuous recording of temperature–humidity index, feed availability, and water access would enable estimation of genotype-by-environment interactions. Repeated sampling across the day would further capture stress-induced disruption of metabolic and microbial rhythms [7].

Phenotyping and sampling. Robust phenotyping should combine standardized methane measurements with feed intake, growth, wool or milk production, and feed-efficiency traits such as residual feed intake. Rumen samples should be collected for microbial and metabolomic profiling, while blood samples should be used to characterize systemic metabolic and oxidative-stress responses under matched sampling conditions.

Molecular layers. Host genomic variation should be assessed using SNP arrays or whole-genome sequencing, while rumen microbial profiles can be generated using scalable approaches such as RE-RRS with reference-free analytical strategies. Targeted VFA analysis, untargeted metabolomics, milk fatty acids, and mid-infrared spectroscopy should be integrated where appropriate to identify scalable biomarkers [4,21,26].

Analysis and validation. Prediction frameworks should combine interpretable mixed models, incorporating genomic, microbial, and metabolomic relationship matrices, with machine-learning approaches capable of detecting nonlinear interactions. Environmental variables should be explicitly incorporated to estimate genotype-by-environment effects. Models should undergo forward validation in subsequent cohorts and external validation across breeds or regions. Finally, candidate microbial and metabolic biomarkers should undergo functional validation to distinguish biological mechanisms from statistical associations. Priority should go to functional validation, such as microbial intervention or inoculation trials and supplementation studies aimed at candidate taxa, to test candidate biomarkers causally.

## 12. Conclusions

Methane traits in sheep are heritable and repeatable, supporting selective breeding for lower emissions. Combining host genomic, rumen microbial, and metabolomic information has produced preliminary improvements in prediction, including modest gains in a 99-sheep proof-of-concept study [10]. Larger microbiome studies and emerging neural-network models provide additional evidence of predictive value [4,26,46], but their results do not establish reliable performance across breeds, diets, and climatic conditions. Low methane yield, feed efficiency, productivity, and resilience remain distinct objectives that must be evaluated together. Evidence under heat stress is limited, and the stability of multi-omics predictions across environments remains insufficiently tested. Progress requires longitudinal, multi-breed studies with standardized phenotyping, explicit environmental measurements, and independent validation in the systems where predictions will be used. Candidate taxa and metabolites should be described as associations or potential predictors until functional and external validation support stronger claims. Multi-omics approaches should complement direct methane measurement, which remains essential for calibration and benchmarking.

## Figures and Tables

**Table 3:** Candidate metabolites and protein stress markers relevant to methane, energy metabolism, and climate stress in sheep.

Metabolite/Class	Compartment	Association	Interpretation	Evidence Setting and References
Propionate	Rumen	Low methane	Hydrogen sink; energetically efficient VFA	Sheep-rumen in vitro study [28]
Acetate/propionate ratio	Rumen	Higher ratio, higher methane	Acetate fermentation releases hydrogen	[21]
Butyrate/valerate	Rumen	Fermentation pattern; muscle and meat-quality responses	Methane-related fermentation hypothesis [20]; separate lamb supplementation evidence for productivity [29]	[20,29]
Branched-chain fatty acids	Milk	Low-methane line	Microbial-membrane origin; indirect community signal	[21]
Polyunsaturated fatty acids	Milk	Low-methane line	Altered biohydrogenation	[21]
Saturated fatty acids	Milk	High in high-methane line	Fermentation-linked lipid profile	[21]
Cortisol; steroid pathway metabolites	Rumen/seminal metabolomic profiles	Heat stress	HPA-axis activation under thermal load	Heat-stressed Hu rams [7]
Kaempferol; L-tryptophan	Rumen/seminal	Downregulated under heat	Linked to oxidative and reproductive function	Heat-stressed Hu rams [7]
HSP60/HSP70/HSP90 (proteins, not metabolites)	Serum	Heat stress	Cellular heat-shock and oxidative response	Heat-stressed lambs [8]; protein stress markers, not methane predictors

Abbreviations: HPA, hypothalamic–pituitary–adrenal; HSP, heat shock protein; VFA, volatile fatty acid.

**Table 4:** Multi-omics data types, analytical methods, advantages, and limitations.

Data Layer	Typical Method	Advantages	Limitations
Host genome	SNP array; GWAS; GBLUP	Heritable, transmissible; measured once per animal	Explains limited variance for moderately heritable methane; few causal variants known in sheep
Rumen microbiome	16S; shotgun metagenomics; RE-RRS	Captures fermentation machinery; scalable and cheap (RE-RRS); reference-free improves prediction	Diet- and time-sensitive; partly transient; genus-level resolution can obscure function
Metatranscriptome	RNA-seq	Shows active expression rather than mere presence	Costly; RNA labile; snapshot in time
Rumen metabolome	GC/LC-MS; NMR	Close to phenotype; direct VFA read-out	Platform-dependent; single-timepoint; invasive sampling
Blood/milk metabolome	LC-MS; MIR of milk	Minimally invasive; milk proxies scalable	Sheep-specific methane links sparse; calibrated against imperfect references
Phenotype & environment	Intake, growth, methane, THI	Defines the selection target and G × E	Methane methods not standardized; environment often unrecorded
Integration/modeling	GBLUP relationship matrices; random forest; gradient boosting; Bayesian; SEM; deep learning	Combining layers may improve prediction; structural models can evaluate specified causal hypotheses under explicit assumptions	High dimensionality and limited independent validation; nonlinear methods have emerging sheep evidence [46], but climate transferability is untested

Abbreviations: GBLUP, genomic best linear unbiased prediction; GC, gas chromatography; GWAS, genome-wide association study; LC-MS, liquid chromatography–mass spectrometry; MIR, mid-infrared spectroscopy; NMR, nuclear magnetic resonance; RE-RRS, restriction-enzyme reduced-representation sequencing; RNA-seq, RNA sequencing; SEM, structural equation modeling; SNP, single-nucleotide polymorphism; THI, temperature–humidity index; VFA, volatile fatty acid.

**Table 5:** Proposed predictive model framework for low-methane, high-feed-efficiency, climate-resilient sheep.

Component	Specification	Rationale/Source	
Target traits	Methane yield; RFI/feed conversion; production (growth, wool, milk); resilience index	Multi-objective, not methane alone	[3,6]
Input, genomic	Ovine SNP genotypes	Heritable component	[24]
Input, microbial	RE-RRS rumen metagenome, reference-free	Scalable proxy; improves accuracy	[4,26]
Input, metabolomic	Rumen VFA; milk fatty acids/MIR where lactating	Close to phenotype; low-cost proxy	[21]
Input, phenotypic	Intake, growth, methane or validated proxy	Defines RFI and calibrates proxies	[23]
Input, environmental	THI, feed and water availability (continuous)	Enables G × E estimation	[7]
Core model	GBLUP with genomic + microbial + metabolomic relationship matrices	Interpretable variance partition	[10]
Validation	Forward (later cohort) + external (breed/region) + cross-method	Guards against overfitting and G × E failure	[4,5,23,26]

Abbreviations: GBLUP, genomic best linear unbiased prediction; MIR, mid-infrared spectroscopy; RE-RRS, restriction-enzyme reduced-representation sequencing; RFI, residual feed intake; SNP, single-nucleotide polymorphism; THI, temperature–humidity index; VFA, volatile fatty acid.

## Data Availability

No new data were created or analyzed in this study. Data sharing is not applicable to this article.
